# Computer Methods for Automatic Locomotion and Gesture Tracking in Mice and Small Animals for Neuroscience Applications: A Survey

**DOI:** 10.3390/s19153274

**Published:** 2019-07-25

**Authors:** Waseem Abbas, David Masip Rodo

**Affiliations:** Multimedia and Telecommunications Department, Universitat Oberta de Catalunya, 08018 Barcelona, Spain

**Keywords:** locomotion tracking, gesture tracking, behavioral phenotyping, automated annotation, neuroscience, machine learning

## Abstract

Neuroscience has traditionally relied on manually observing laboratory animals in controlled environments. Researchers usually record animals behaving freely or in a restrained manner and then annotate the data manually. The manual annotation is not desirable for three reasons; (i) it is time-consuming, (ii) it is prone to human errors, and (iii) no two human annotators will 100% agree on annotation, therefore, it is not reproducible. Consequently, automated annotation for such data has gained traction because it is efficient and replicable. Usually, the automatic annotation of neuroscience data relies on computer vision and machine learning techniques. In this article, we have covered most of the approaches taken by researchers for locomotion and gesture tracking of specific laboratory animals, i.e. rodents. We have divided these papers into categories based upon the hardware they use and the software approach they take. We have also summarized their strengths and weaknesses.

## 1. Introduction

Neuroscience has found an unusual ally in the form of computer science which has strengthened and widened its scope. The wide availability and easy-to-use nature of video equipment have enabled neuroscientists to record large volumes of behavioral data of animals and analyze them from the neuroscience perspective. Traditionally, neuroscientists would record videos of animals they wanted to study and then annotate the video data manually. Normally, this approach is reasonable if the video being annotated is not large, but the bigger the volume of recorded data gets, the more inconvenient, tiresome, erroneous and slow the manual annotation becomes. Moreover, the annotations made by human annotators are not perfectly reproducible. Two annotations of the same sample done by two different persons will likely differ from each other. Even the annotation done for the same sample at different times by the same person might not be the same. All of these factors have contributed to the demand for a general-purpose automated annotation approach for video data. For behavioral phenotyping and neuroscience applications, researchers are usually interested in gesture and locomotion tracking. Fortunately, computer science has an answer to this problem in the form of machine learning and computer vision-based tracking methods. The research in this area is still not mature, but it is receiving a lot of attention lately. The primary motivation for automated annotation is the reproducibility and ability to annotate large volumes of data in a practical amount of time.

Some researchers approach this problem by treating a video as a sequence of still images and then applying computer vision algorithms to every frame without considering their temporal relationships [1,2]. Some of the researchers include temporal information to some extent while others use the assistance of additional hardware [3,4,5]. The general framework is similar. Animals (mice/rats/insects) are kept in a controlled environment, either restrained or free where the lighting and illumination can be manipulated. To acquire the video data, single or multiple video cameras are installed. These might be simple video cameras or depth/IR cameras. There might be some additional accessories installed such as physical markers or body-mounted sensors [6,7,8,9]. In this article, we review the state of the approaches for rodents’ gesture and locomotion tracking. Nevertheless, we do not restrict the review only to previous works which focus only on rodents, but we include similar approaches that could be easily ported to this particular case (typically other small mammals and insect monitoring applications).

## 2. Problem Statement

**Figure 1:**
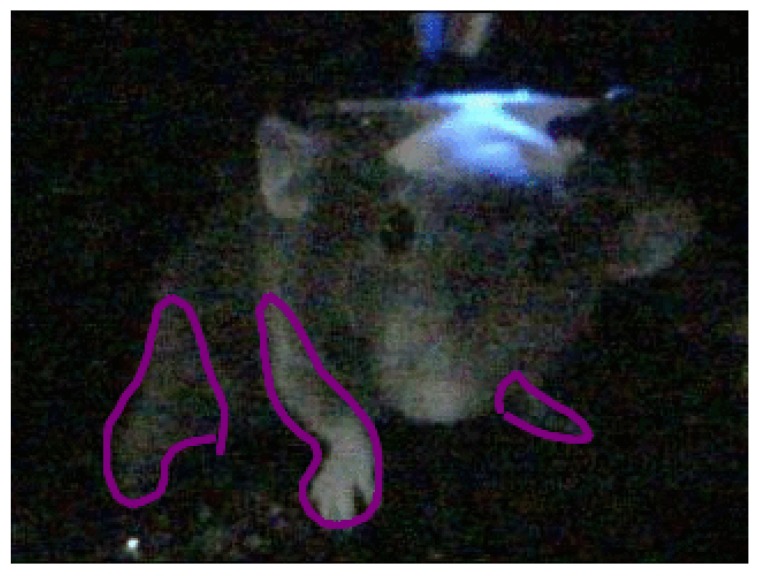
Frontal view of a mouse with its moving limbs marked.

**Figure 2:**
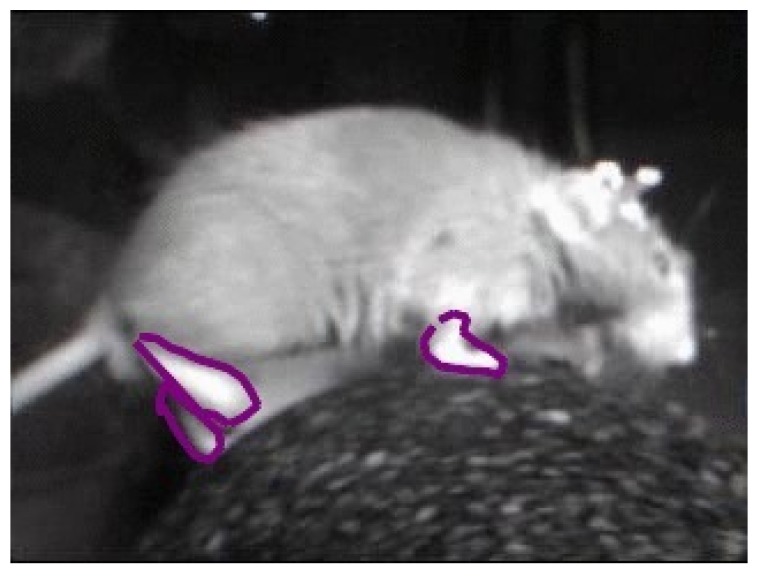
Lateral view of a mouse with its moving limbs marked.

Behavioral phenotyping depends upon the annotated activity of rodents/small animals. We can identify the activity when we see how the rodents/small animals move, behave and act over an extended periods of time. One of the many proposed approaches is to track the limb movements of the rodents and convert them into quantifiable patterns. Limb tracking can be either achieved by recording the limbs from the frontal, lateral, top or bottom view. Cases shown in Figure 1 and Figure 2 are typical examples of activity tracking in rodents and small animals. They present the following challenges:

1. Spatial resolution in most consumer-grade video cameras is not sufficient for effective tracking when the temporal resolution increases. Usually, cameras increase the frames per second ratio by decreasing the image resolution.
2. Limbs might move faster at one point in time while they might be stationary at another point in time, rendering the development of uniform motion model impossible.
3. The limbs might overlap with each other or other body parts, therefore, presenting occlusions.
4. Some settings require specific lighting conditions, which may make automated gesture recognition more difficult.

## 3. Motion Tracking Principles in Videos

Videos are sequences of images/frames. When displayed with sufficient frequency, they will appear as continuous content to the human eye. Therefore, all the image processing techniques can be applied to the individual video frames [10,11]. Moreover, the contents of two consecutive frames are often closely related, thus, making object and motion tracking possible in videos. Motion detection/object tracking in videos is done by detecting objects in individual frames. It involves monitoring an object’s shape and motion trajectory in every frame. This is achieved by solving the temporal correspondence problem, to match a region in successive frames of a video sequence [12,13,14].

Motion detection provides additional information for detection and tracking. Most of the state-of-the-art methods involve single or multiple techniques for motion detection. For the sake of clarity in this survey, we divide these approaches in background subtraction/temporal differencing and statistical/learning-based approaches.

### 3.1. Background Subtraction-Based Approaches

Commonly used for motion segmentation in static scenes, background subtraction attempts to detect and track motion by subtracting the current image pixel-by-pixel from a reference/background image. The pixels which yield a difference above a threshold are considered as foreground. The creation of the background image is known as background modeling. Once the foreground pixels are classified, some morphological post-processing is done to enhance the detected motion regions. Different techniques for background modeling, subtraction and post-processing result in different approaches for the background subtraction method [15,16,17,18,19].

In temporal differencing, motion is detected by taking pixel-by-pixel difference of consecutive frames (two or three). It is different from background subtraction in the sense that the background or reference image is not stationary. It is mainly used in scenarios involving a moving camera [20,21,22,23,24].

### 3.2. Statistical and Learning-Based Approaches

Some methods distinguish between foreground and background keeping and updating statistics of the foreground and background pixels. Foreground and background pixels are differentiated by comparing pixel statistics with that of the background model. So, in essence, motion tracking is achieved by tracking the statistical models of foreground (object) and the background in each video frame. This approach is stable in the presence of noise, illumination changes and shadows [25,26,27,28,29,30,31,32,33]. Some approaches employ optical flow to track the apparent motion and then use it to predict the position/pose in the next frames. Optical flow is the distribution of apparent velocities/ movement of brightness patterns in an image [34,35,36,37,38,39,40]. In some cases, the optical flow-based prediction is further reinforced by introducing a learning element, algorithms are trained either to predict the position of the object in successive frames or pose of a person/animal or detect a specific object in each frame. Some of these learning-based approaches do not rely on the explicit estimation of optical flow at all, instead, they try to solve this problem by learning how the object looks and then tracking similar objects in every frame or by learning how the object moves [41,42,43].

## 4. Major Trends

Motion tracking for neuroscience applications is not formally different from general motion tracking; therefore, all the motion tracking techniques can be applied to it in one way or the other. Although the general idea is the same, the environment for such type of motion tracking can be different from general-purpose tracking. A typical setup for neuroscience applications includes a closed environment (either a room or a box), video cameras, the animal and control systems. The animal can either be restrained or freely behaving. There might multiple cameras recording the motion from different angles. For this survey, we will go through all those cases which involve motion tracking (especially limbs tracking, head tracking and gesture tracking) of laboratory animals for behavioral phenotyping or medical assessment purposes. It is to be noted that research on gesture tracking/pose estimation for humans has seen significant improvements in recent years, those techniques cannot be applied as they are to gesture tracking in rodents and small animals for the following reasons:

1. Human gait parameters and motion patterns are inherently different than those of four-legged animals/rodents/lab animals.Human gait can be solely represented by an inverted pendulum model while in rats/four-legged animals, the inverted pendulum model represents only a percentage of the gait (up to 70% according to some researchers). Moreover, the degree of freedom for human gait is different than that of four-legged animals. [44,45,46,47].
2. Gesture tracking techniques developed for humans are mostly optimized for the environments in which humans dwell, therefore, they can’t be directly imported for lab environments.
3. Human subjects do not need to be trained to perform a supervised task. For example, let’s say a neuroscientist wants to investigate the effect of a certain neurophysiological regime on physical activity, he/she can simply ask the test subject to either walk or exercise. The same cannot be said for small animals/rodents. They need to be trained on a treadmill, therefore, the tracking methods developed for humans might not have the same efficiency for rodents.
4. For reliable behavioral phenotyping, the gesture tracking/pose estimation should be highly accurate, therefore, they often need more fine-tuning.

Although techniques developed for human gesture tracking/pose estimation cannot be applied as they are to gesture tracking in rodents and small animals, many of the studies mentioned in this survey either take inspiration from those techniques or built their solutions based upon them.

**Figure 3:**
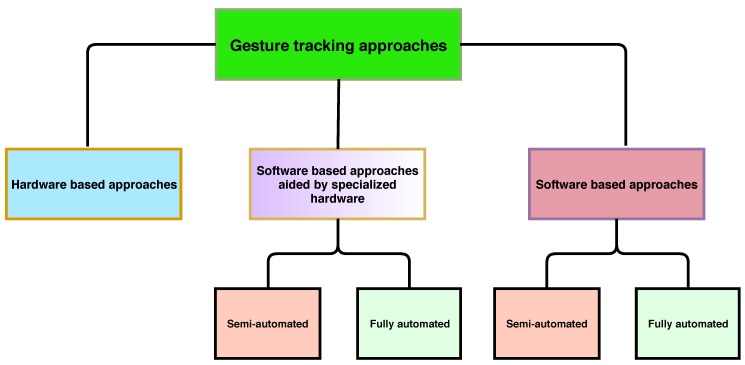
Categories and their hierarchy of the approaches covered in this survey paper.

Based on their intended use and nature, we have divided the approaches according to the hierarchy outlined in Figure 3.

## 5. Hardware Based Methods

LocoWhisk is a commercial solution that proposes to quantify and track locomotion by tracking the whiskers’ movements using a specialized hardware setup [48]. The setup is comprised of high-speed cameras, a pedobarograph, and infrared lighting. Open-source image processing techniques are then used to track the infra-red illuminated whiskers. The inventors behind this solution haven’t provided any objective evaluation of how effective is their solution in tracking whisking movements. The solution is not compatible with any existing equipment, therefore, it has to be bought and installed from scratch. Also, since they haven’t provided the details into the image processing pipeline, we cannot compare and validate its effectiveness.

Kain et al. [49] proposed an explicit hardware-based leg tracking method for automated behavior classification in Drosophila flies. The fly is made to walk on a spherical treadmill. Dyes which are sensitive to specific wavelengths of light are applied to its legs and then the leg movement is recorded by two mounted cameras. This way, 15 gait features are recorded and tracked in real-time. This approach has the appeal for real-time deployment but it cannot be generalized to any limb tracking application because it needs a specific hardware setup. Moreover, being heavily dependent on photo-sensitive dyes decreases its robustness. Also, the flies have to walk on a spherical treadmill for this method to be effective which is not always easy since it is very hard to train the flies.

Snigdha et al. [50] proposed 3D tracking of mice whiskers using optical motion capture hardware. The 3D tracking system (Hawk Digital real-time System, Motion Analysis Corp., Santa Rosa, CA, USA) is composed of two joint cameras and the Cortex analysis software (Motion Analysis, CA, USA). The whiskers are marked with retro-reflective markers and their X, Y, and Z coordinates are digitized and stored along with video recordings of the marker movements. The markers are made from a retro-reflective tape backed with adhesive (Motion Analysis Corp., Santa Rosa, CA, USA) and fastened onto the whiskers using the tape’s adhesive. Markers were affixed to the whisker at a distance of about 1 cm from the base. Reliable 3D tracking requires a marker to be visible at all times by both cameras. This condition can be satisfied in head-fixed mice where the orientation of the mouse to the cameras remains fixed. The system was connected to a dual processor Windows-based computer for data collection. The proposed tracking framework is easy to install and computationally cheap. The hardware components are not also high end and expensive, therefore, this system can be set up relatively cheaply. But like other hardware-assisted frameworks, it also needs specialized hardware and thus, it isn’t very scalable and portable. Moreover, for reliable tracking, the retro-reflective markers should be visible to the cameras at all times, therefore, it cannot handle occlusions and thus, it is not robust. Also, since the method is invasive, it might affect the mice behavior, therefore, rendering the behavioral analysis results skewed.

Scott Tashman et al. [51] proposed a bi-plane radiography assisted by static CT scan-based method for 3D tracking of skeletons of small animals. The high-speed biplane radiography system consists of two 150 kV X-ray generators optically coupled to synchronized high-speed video cameras. For static radiostereometric analysis, they implanted a minimum of three radio-opaque bone markers per bone to enable accurate registration between the two views. The acquired radiographs are first corrected for geometric distortion. They calculated ray-scale weighted centroids for each marker with sub-pixel resolution. They tested this system on dogs and reported an error of 0.02 mm when inter-marker distance calculated by their system was compared to the true inter-marker distance of 30 mm. For dynamic gait tracking, this system is reported to be very accurate but the accuracy comes at a cost, the system is expensive and need dedicated hardware. Also, since the system includes specialized hardware, it is not easy to operate. Moreover, since the marker implantation is invasive, it can alter the behavior of animals being studied.

Harvey et al. [52] proposed an optoelectronic based whisker tracking method for head-fixed rats. In the proposed method, the rat’s head is fixed to a metal bar protruding from the top of the restraining device. Its paw rests on a micro switch that records lever presses. A turntable driven by a stepper motor rotates a single sphere/cube into the rat’s “whisking space”. The whiskers are marked to increase the chances of detection. The movements of a single whisker are detected by a laser emitter and an array of CCD detectors. Once the data is recorded, a single whisker is identified manually which serves as a reference point. As the article is more focused on whisking responses of rodents to external stimuli, they have not reported the whiskers’ detection and tracking accuracy. R. Bermejo et al. [53] reported a similar approach for tracking individual whiskers. They restrained the rats and then used a combination of CCDs and laser emitters. The rats were placed in such a way that their whiskers blocked the path of the laser, casting a shadow over CCDs, thus, registering the presence of a whisker which can be tracked by tracking the voltage shifts on CCD array. They also have not reported tracking accuracy. Both of these methods need the whiskers to be visible at all time, therefore, these approaches cannot perform well in the case of occlusions. Moreover, the head of the rats need to be fixed, so they cannot be studied while behaving freely. Also, apart from the need for specialized hardware, the system needs the user to initialize tracking, so it is not completely automated.

Kyme et al. [54] proposed a marker-assisted hardware-based method for head motion tracking of freely behaving and tube-bounded rats. They glued a marker with a specific black and white pattern to the rat’s head. Motion tracking was performed using the Micron-Tracker S×60 (ClaronTech. Inc., Toronto, ON, Canada), a binocular-tracking system that computes a best-fit pose of printed markers in the field of measurement [55]. The authors have reported accurate tracking for more than 95% of the time in the case of tube-bounded rats and similar performance for freely behaving rats if the tracking algorithm is assisted 10% of the time. The system is simple and effective for tube-bound rats and can be operated easily. But the approach has one major drawback; it can only be used in a very specific setting. It requires a specialized setup and it needs to glue external markers to the test subject’s head, which might affect its behavior. Moreover, the same authors have used the Micron-Tracker based approach for synchronizing head movements of a rat with positron emission tomography scans of their brains and have reported that the marker-assisted tracking method was able to synchronize the head movements with scan intervals with an error of less than 10 ms [56].

Pasquet et al. [57] proposed a wireless inertial sensors-based approach for tracking and quantifying head movements in rats. The inertial measurement unit (IMU) contains a digital 9-axis inertial sensor (MPU-9150, Invensense, San Jose, CA, USA) that samples linear acceleration, angular velocity and magnetic field strength in three dimensions, a low-power programmable microcontroller (PIC16, Microchip, Chandler, AZ, USA) running a custom firmware and a Bluetooth radio, whose signal is transmitted through a tuned chip antenna. This system was configured with Labview for data acquisition and the analysis was done in R. The sensors record any head movements by registering the relative change in acceleration. Since the sensors record data in nine axes, the method is used to detect events in rat’s behavior based on head movements. The authors have reported a detection accuracy of 96.3% and a mean correlation coefficient of 0.78 ± 0.14 when the recorded data is compared for different rats (n = 19 rats).Since the proposed system records a head’s acceleration, angular velocity and magnetic field strength in all three dimensions, this opens up the possibility of using this dataset for high-end learning algorithms for behavioral classification. Moreover, since the dataset is based on well-studied physical phenomena (acceleration and velocity), it can also be used to develop deterministic models of the head’s movements. The reported performance figures are very good in terms of event detection and consistency but the system can only be used to track head movements. Also, the system requires specialized hardware which limits its portability. Since the method needs an inertial sensor to be attached to the head of the rats, it is invasive and therefore, can alter the rat’s behavior.

Hamers et al. proposed a specific setup based on inner-reflecting plexiglass walkway [58]. The animals traverse a walkway (plexiglass walls, spaced 8 cm apart) with a glass floor (109, 3, 15, 3, 0.6 cm) located in a darkened room. The walkway is illuminated by a fluorescent tube from the long edge of the glass floor. For most of the way, the light travels internally in the glass walkway, but when some pressure is applied, for example by motion of a mouse, the light escapes and is visible from outside. The escaped light, which is scattered from the paws of the mouse, is recorded by a video camera aimed at a $45^{\circ }$ mirror beneath the glass walkway. The video frames are then thresholded to detect bright paw prints. The paws are labeled (left, right, front, hind). The system can extrapolate a tag (label of the footprint) to the bright areas in the next frame which minimizes the need for user intervention but in some cases, user intervention becomes necessary. The authors haven’t reported paw detection/tracking performance. The system is reliable for paw-tracking but the required setup makes its wide-scale application less likely, therefore, it can only be used for one specific purpose.

## 6. Video Tracking Aided by Hardware

### 6.1. Semi-Automated

Dorman et al. [59] conducted a comparative study of two commercially available hardware-assisted gait analysis systems; DigiGait and TreadScan. The $DigiGait^{TM}$ imaging system uses a high-speed, 147 frames-per-second video camera mounted inside a stainless steel treadmill chassis below a transparent treadmill belt to capture ventral images of the subject. The treadmill is lit from the inside of the chassis by two fluorescent lights and overhead by one fluorescent light. The $TreadScan^{TM}$ imaging system uses a high-speed, 100 frames-per-second video camera adjacent to a translucent treadmill belt to capture video reflected from a mirror mounted under the belt at $45^{\circ }$. Images are automatically digitized by $DigiGait^{TM}$ and $TreadScan^{TM}$ systems. $DigiGait^{TM}$ videos are manually cropped and imported, and then automatically analyzed. The software identifies the portions of the paw that are in contact with the treadmill belt in the stance phase of stride as well as tracks the foot through the swing phase of the stride. Measures are calculated for 41 postural and kinematic metrics of gait. The authors found that $DigiGait^{TM}$ system consistently measured significantly longer stride measures than $TreadScan^{TM}$. Both systems’ measures of variability were equal. Reproducibility was inconsistent in both systems. Only the $TreadScan^{TM}$ detected normalization of gait measures and the time spent on analysis was dependent on operator experience. $DigiGait^{TM}$ and $TreadScan^{TM}$ have been particularly well received in neurophysiological research [60,61,62,63,64,65,66,67,68,69,70].

Cleversys Inc. (http://cleversysinc.com/CleverSysInc/) introduced a commercial solution for gait analysis in rodents, called GaitScan [71]. GaitScan system records videos of the rodent running either on a transparent belt treadmill or on a clear free-walk runway. The video of the ventral (underside) view of the animal is obtained using a high-speed digital camera. The video essentially captures the footprints of the animal as they walk/run. GaitScan software can work with videos taken from any treadmill or runway device that allows the capture of its footprints on any video capturing hardware system with a high-speed camera. The accompanying software lets the user track multiple gait parameters which can be later used for behavioral phenotyping. This solution has also been used in multiple studies [72,73,74,75,76].

TrackSys ltd. (http://www.tracksys.co.uk/) introduced two commercial systems for rodents’ motor analysis. One system is called ’ErasmusLadder’. The mouse traverses a horizontal ladder between two goal boxes. Each rung of the ladder contains a touch-sensitive sensor. These sensors allow the system to measure numerous parameters relative to motor performance and learning such as step time and length, missteps, back steps and jumps [77]. It has been used in multiple studies [78,79,80,81,82]. Its tracking performance hasn’t been reported by its manufacturer. The other system is called ’CatWalk’ [83]. It is comprised of a plexiglass walkway that can reflect light internally. When the animals’ paws touch the glass, the light escapes as their paw print and is captured by a high-speed camera mounted beneath the walkway. It can be used to quantify several gait parameters such as pressure, stride length, swing and stance duration. Multiple researchers have used ’CatWalk’ in gait analysis [84,85,86,87,88].

Knutsen et al. [89] proposed the use of overhead IR LEDs along with video cameras for head and whisker tracking of unrestrained behaving mice. The overhead IR LEDs are used to flash IR light onto the mouse head which is reflected from its eyes. The reflected flash is recorded by an IR camera. In the first few frames of every movie, a user identifies a region-of-interest (ROI) for the eyes which encircles a luminous spot (reflection from the eye). This luminous spot is tracked in subsequent frames by looking for pixels with high luminosity in the shifted ROI. Once eyes are located in every frame, they are used to track head and whiskers in intensity videos. First, a mask averaged over the frames containing no mice is subtracted from the frame. Then user-initiated points are used to form whisker shaft by spline interpolation. For the next frame, sets of candidate points are initiated and shaft from current frames is convolved with candidate shafts from the next frame to locate the set of points most likely being a whisker. Although the pipeline has no temporal context involved, yet it is quite effective in whisker tracking with a high Pearson correlation between ground truth and tracked whisker shafts. The downside of this approach is the need for high-speed videos and additional IR hardware. Moreover, since the whisker tracking is dependent upon the accurate detection of eyes in every frame for finding the region of interest which contains the head, any flashes onto the IR camera or any occlusions of the eyes can result in a considerable deviation in whisker tracking.

Gravel et al. [90] proposed a tracking method assisted by an X-Ray area scan camera for gait parameters of rats walking on a treadmill. The system consists of a Coroskop C arm X-ray system from Siemens, equipped with an image intensifier OPTILUX 27HD. The X-Ray system is used to detect fluoroscopic markers placed on hind limbs of the rat. A high-speed area scan camera from Dalsa (DS-41-300K0262), equipped with a C-mount zoom lens (FUJINON-TV, H6X12.R, 1:1.2/12.5–75) mounted on the image intensifier is used for video acquisition and a computer is used to overlay the detected markers on the video. The treadmill with the overlaying box is placed on a free moving table and positioned near the X-ray image intensifier. The X-ray side view videos of locomotion are captured while the animal walks freely at different speeds imposed by the treadmill. The acquired video and marker data are processed in four steps; correction for image distortion, image denoising and contrast enhancement, frame-to-frame morphological marker identification and statistical gait analysis. The data analysis process can be run in automated mode for image correction and enhancement however the morphological marker identification is user-assisted. The kinematic gait patterns are computed using a Bootstrap method [91]. After multiple Monte Carlo runs, the authors have reported consistent gait prediction and tracking with confidence of 95%. They have compared the performance of the proposed system with manual marker annotation by a user by first manually processing 1 h 30 min of data and then processing only 12 min data by the system assisted by the same user. They have reported only 8% deviation in gait cycle duration, therefore, claiming a 7-fold decrease in processing time with acceptable loss in accuracy. The system is robust for gait pattern analysis, it can track multiple gait parameters, therefore, making complex behavioral classification possible. However, the system is still not scalable and portable because it relies on dedicated hardware. The system is not fully automated as well as it relies upon continuous user assistance. Moreover, the system needs physical markers painted on the limbs, therefore, it cannot work reliably in a situation where painting markers is not an option.

John et al. [92] proposed a semi-automated approach for simultaneously extracting three-dimensional kinematics of multiple points on each of an insect’s six legs. White dots are first painted on insect’s leg joints. Two synchronized video cameras placed under the glass floor of the platform are used to record video data at 500 frames per second. The synchronized video data is then used to generate 3D point clouds for the regions of interest by triangulation. The captured video frames are first subtracted from a background frame modeled by a Gaussian mean of 100 frames with no insects. After image enhancement, a user defines the initial tracking positions of leg joints in a 3D point cloud which are then tracked both in forward and backward direction automatically. The user can correct any mismatched prediction in any frame. The authors have reported a tracking accuracy of 90% when the user was allowed to make corrections in 3–5% of the frames. The proposed approach is simple in terms of implementation, accurate in terms of spatial and temporal resolution and easy to operate. Also, the proposed method produced a rich dataset of insects’ legs kinematics, therefore, making complex behavioral analysis possible. However, it needs constant user assistance and does not have any self-correction capability.

### 6.2. Completely Automated

#### 6.2.1. Background Subtraction-Based Approaches

Akihiro Nakamura et al. [93] proposed a depth sensor-based approach for paw tracking of mice on a transparent floor. The system is composed of an open-field apparatus, a Kinect sensor, and a personal computer. It captures the subject’s shape from below using a low-cost infrared depth sensor (Microsoft Kinect) and an opaque infrared-pass filter. The open field is a square of 400mm×400mm and the height of the surrounding wall is 320 mm. The Kinect device is fixed 430 mm below the floor so that the entire open-field area can be captured by the device. For the experiment in the opaque conditions, the floor of the open field was covered with tiled infrared-pass filters (FUJIFILM IR-80 (Fuji Film, Tokyo, Japan)), which are commonly used in commercial cameras. The depth maps, consisting of 320×240 depth pixels, are captured at 30 frames per second. The tracking algorithm has four steps; pre-processing, feature-point extraction, footprint detection, and labeling. During pre-processing, the subject’s depth information is extracted from the raw depth map by applying background subtraction to the raw depth map. The noise produced by pre-processing steps is removed by morphological operations. AGEX algorithm [94] is used for feature extraction after pre-processing. Center of mass of AGEX point clouds is used for paw detection and labeling. All those pixels whose Euclidean distance is lower than a threshold from the center of mass are considered to be member pixels of the paws. This framework offers the benefits of low computational cost and easy-to-install system. The proposed system can also be used in real-time. However, it is not robust. It can be used only for paw tracking in a specific setting. Moreover, it cannot be used for other gesture tracking measures, such as head or whiskers tracking.

César S. Mendes et al. [95] proposed an integrated hardware and software system called ’MouseWalker’ that provides a comprehensive and quantitative description of kinematic features in freely walking rodents. The MouseWalker apparatus is comprised of four components: the fTIR floor and walkway wall, the supporting posts, the $45^{\circ }$ mirror, and the background light. A white LED light strip for black and white cameras or a colored LED light strip for color cameras is glued to a 3/8-inch U-channel aluminum base LED mount. This LED/aluminum bar is clamped to the long edges of a 9.4-mm (3/8-inch) thick piece of acrylic glass measuring 8 by 80 cm. A strip of black cardboard is glued and sewn over the LED/acrylic glass contact areas. To build the acrylic glass walkway, all four sides were glued together with epoxy glue and cable ties and placed over the fTIR floor. Videos are acquired using a Gazelle 2.2-MP camera (Point Grey, Richmond, VA, Canada) mounted on a tripod and connected to a Makro-Planar T 2/50 lens (Carl Zeiss, Jena, Germany) at maximum aperture (f/2.0) to increase light sensitivity and minimize depth of field. The ’MouseWalker’ program is developed and compiled in MATLAB (The Mathworks, MA, USA) [96]. The body and footprints of the mouse are distinguished from the background and each other based on their color or pixel intensity. The RGB color of the mouse body and footprints are user-defined. The tail is identified as a consecutive part of the body below a thickness threshold. Three equidistant points along the tail are used to characterize tail curvature. The head is defined by the relative position of the nose. The center and direction of the head are also recorded along with the center of the body without the tail and its orientation. A body “back” point is defined as the point which is halfway between the body center and the start of the tail. For the footprints of the animal, the number of pixels within a footprint, as well as the sum of the brightness of these pixels, are stored by the software. The ’MouseWalker’ can be used to track speed, steps frequency, swing period and length of steps, stance time, body linearity index footprint clustering and leg combination indexes: no swing, single-leg swing, diagonal-leg swing, lateral-leg swing, front or hind swing, three-leg swing, or all-legs swing (unitless). The system is quite robust; it can track multiple gait parameters and can create a rich dataset which can be used to train advanced learning-based algorithms for behavioral classification. However, the system is not very scalable and portable. The enclosing setup of the mice and the hardware configuration is too specific for portability.

Wang et al. [97] proposed a pipeline for tracking motion and identifying the micro-behavior of small animals based on Microsoft Kinect sensors and IR cameras. This is achieved by employing Microsoft Kinect cameras along with normal video cameras to record movement of freely behaving rodents from three different perspectives. The IR depth images from Microsoft Kinect are used to extract the shape of the rodents by background subtraction. After shape extraction, five pixel-based features are extracted from the resultant blobs which are used for tracking and behavior classification by Support Vector Machines. Although the pipeline is not exclusively used for motion tracking, the idea of using depth cameras is potentially a good candidate for motion tracking as well.

Voigts et al. proposed an unsupervised whisker tracking pipeline aided by the use of IR sensors for selective video capturing [98]. They capture high speed (1000 frames per second) video data by selectively recording those frames which contain the mice. It is achieved by sensing the mice by an IR sensor which then triggers the video camera to start recording. Once the mice leave the arena, the IR sensors trigger the video camera to stop capturing. This selectively-acquired video data is used for whisker tracking. First, a background mask is calculated by averaging over 100 frames containing no mice. This mask is subtracted from every single frame. Then vector fields from each frame that resulted in a convergence of flows on whisker-like structures are generated. These fields are then integrated to generate spatially continuous traces of whiskers which are grouped into whisker splines. This approach is completely unsupervised when it comes to whisker tracking with a rough temporal context as well. Moreover, instead of high speed video acquisition, the on-demand recording when the mice are in the arena cuts the memory requirements and this approach can be exported to other motion/gesture tracking pipelines. However, it is very greedy in terms of computational resources so it cannot be employed in real-time.

Nashaat et al. proposed an automated optical method for tracking animal behavior in both head-fixed and freely moving animals, in real-time and offline [99]. They use a Pixy camera (Charmed labs, Carnegie Mellon University, equipped with a 10–30 mm f1.6 IR lens, controlled by open-source PixyMon software) based system for real-time tracking. The Pixy camera needs the whisker to be painted with a UV sensitive dye. For more detailed tracking, they use the tracking from the Pixy camera to guide the tracking from high definition cameras offline. They haven’t provided tracking results in comparison to ground truth but they have validated their system in different lighting conditions and environments. On one hand, their system does not need high performance and computationally expensive tracking algorithms while on the other hand, their system is not robust because it requires specialized hardware. Also, their approach is invasive because it requires physical markers (UV sensitive dye on whiskers).

#### 6.2.2. Statistical/Learning-Based Approaches

Monteiro et al. [100] took a similar approach to Wang et al. [97] by using Microsoft Kinect depth cameras for video capturing [100]. Instead of using background subtraction, they introduced a rough temporal context by tracking the morphological features of multiple frames. In their approach, the morphological features are extracted frame by frame. Then features from multiple adjacent frames are concatenated to introduce a rough temporal context. A decision tree is then trained from this dataset for automatic behavior classification. The authors have reported a classification accuracy of 66.9% when the classifier is trained to classify four behaviors on depth map videos of 25 min duration. When only three behaviors are considered, the accuracy jumps to 76.3%. Although the introduced temporal context is rough and the features are primitive, the classification performance achieved firmly establishes the usefulness of machine learning in gesture tracking for behavioral classification. Like [100], this approach is also not solely used for motion tracking, but they have introduced a rough temporal context for tracking along with depth cameras which can be beneficial in motion-tracking-only approaches.

Petrou et al. [101] proposed a marker-assisted pipeline for tracking legs of female crickets. The crickets are filmed with three cameras, two mounted above and one mounted below the crickets which are made to walk on a transparent glass floor. Leg joints are marked with fluorescent dyes for better visualization. The tracking procedure is initiated by a user by selecting a marker position in initial frames. The initial tracking is carried out to the next frames by constrained optimization and Euclidean distance between joints of the current frame and the next frame. This pipeline does a decent job in terms of tracking performance as the average deviation between human-annotated ground truth (500 digitized frames) and automatic tracking is 0.5 mm where the spatial depth of the camera is 6 pixel/mm. This approach has the potential to be applied in real-time and the required setup is not too difficult to make. However, since markers have to be painted on the legs of the cricket, it is invasive and thus, it can alter the behavior of subject under study. Moreover, this approach has only been tested on crickets, so we cannot assume that it will work with rodents/small animals.

Xu et al. [102] proposed another marker-assisted tracking pipeline for small animals. In the proposed pipeline, the limbs and joints are first shaved, marked with dyes and then recorded with consumer-grade cameras (200 frames per second). Tracking is then done in three steps which include marker position estimation, position prediction, and mismatch occlusion. The marker position is estimated by correlation in two methods. In one method, normalized cross-correlation between the grayscale region of interest and user-generated sample markers is found. The pixels with the highest correlation are considered as the marker pixels. In the second method, the normalized covariance matrix of marker model and color ROI is used to estimate pixels with the highest normalized covariance values which are considered as marker pixels. Once the marker positions are estimated in the current frame, they are projected to the next frame by polynomial fitting and Kalman filters. For occlusion handling, they assume that a marker position or image background cannot change abruptly, so if there is a sudden change, it must be an occlusion. The approach is simple and scalable enough to be exported to any environment. Moreover, this pipeline has included measures to solve the problem of occlusions as well. However, due to its dependency on markers, it cannot be exported for general purpose motion/gesture tracking. Also, the markers are placed in a very invasive way, therefore, possibly altering the behavior of test subjects.

Hwang et al. [103] followed a similar approach to the one proposed by John et al. [92] but without the use of markers. They used a combination of six-color charge-coupled device (CCD) cameras (Sca640-70fc, BASLER Co., Schiller Park, IL, USA) for video recording of the insects. To capture the diverse motions of the target animal, they used two downward cameras and four lateral cameras as well as a transparent acrylic box. The initial skeleton of the insect was calculated manually, so the method is not completely automated. After the initial skeleton, they estimated the roots and extremities of the legs followed by middle joints estimation. Any errors in the estimation were corrected by Forward And Backward Reaching Inverse Kinematics (FABRIK) [104]. The authors have not reported any quantitative results which might help us to compare it with other similar approaches however they have included graphics of their estimation results in the paper. This paper does not directly deal with motion estimation in rodents, however, given the unique approach to using cameras and pose estimation, it is a worthwhile addition to the research in the field. Since the pipeline tries to solve gesture tracking from a pose estimation perspective, it opens the possibility of using state-of-the-art pose estimation techniques for gesture tracking in rodents/small animals. However, because of its reliance on initial skeleton, the system cannot be exported for general purpose use.

## 7. Video Tracking Methods Mostly Dependent on Software-Based Tracking

In this section, we will focus on those research works that try to solve the locomotion and gesture tracking problem by processing raw and unaided video streams. In this scenario, there is neither specialized hardware installed apart from one or multiple standard video cameras nor physical markers on the mice/animals bodies that can help to track its motion. These works approach the problem from a pure computer vision point of view.

### 7.1. Semi-Automated

#### Background Subtraction-Based Approaches

Gyory et al. [105] proposed a semi-automated pipeline for tracking rat’s whiskers. In the proposed pipeline, videos are acquired with high-speed cameras (500 frames per second) and are first pre-processed to adjust the brightness. The brightness adjusted images are eroded to get rid of small camera artifacts. Then a static background subtraction is applied which leaves only the rat body in the field of view. As whiskers are represented by arcs with varying curvature, a polar-rectangular transform is applied and then a horizontal circular shift is introduced so that whiskers are aligned as straight lines on a horizontal plane. Once the curved whiskers are represented by straight lines, the Hough transform is used to locate them. This approach can be used a starting point by a researcher who wants to experiment with different automated background subtraction methods for gesture tracking/pose estimation but the approach is too weak itself and not robust enough to be considered for any future improvements. The reported computational cost is high (processing speed of 2 fps). Also, it works on high-speed videos (>500 fps). It is highly sensitive to artifacts and it cannot take care of occlusion, dynamic noise, and broken whisker representation.

### 7.2. Completely Automated

#### 7.2.1. Background Subtraction-Based Approaches

Da Silva et al. [106] conducted a study on the reproducibility of automated tracking of behaving rodents in controlled environments. Rats in a circular box of 1 m diameter with 30 cm walls. The monitoring camera was mounted in such a way that it captured the rodents from top view while they were behaving. They used a simple thresholding algorithm to determine pixels belonging to the rodent. Although the method is rudimentary as compared to state-of-the-art, the authors have reported a Pearson correlation of r=0.873 when they repeated the same experiment at different ages of the animals, thus, validating its reproducibility. However, this setup can only be used to track the whole body of rodents, it cannot identify micro-movements such as limbs motion.

Leroy et al. [107] proposed the combination of transparent Plexiglas floor and background modeling-based motion tracking. The rodents are made to walk on a transparent plexiglass floor illuminated by fluorescent light and is recorded from below. A background image is taken when there is no mouse on the floor. This background image is then subtracted from every video frame to produce a continuously updating mouse silhouette. The tail of the mouse is excluded by an erosion followed by dilation of the mouse silhouette. Then the center of mass of the mouse is calculated which was tracked through time to determine if the mouse is running or walking. Since the paws are colored, color segmentation is used to isolate paws from the body. The authors have reported a maximum tracking error of 4mm±1.9 and a minimum tracking error of 2 mm ±1.6 when 203 manually annotated footprints are compared to their automatic counterparts. This approach has the advantages of easy-installation and simplicity, yet it can track the mouse from only one side. Moreover, being dependent on colored paws, this approach cannot be exported for general purpose gesture tracking/pose estimation.

Nathan et al. [108] proposed a whisker tracking method for mice based on background subtraction, whisker modeling, and statistical approaches. The heads of the mice are fixed, so they are not behaving freely. They use a high-speed camera with a shutter speed of 500 frames per second. To track whiskers, an average background image is modeled from all the video frames and then subtracted from every single frame. Afterward, pixel-level segmentation is done to initiate candidate sites by looking for line like artifacts. Once the candidate boxes are initiated, they are modeled by two ellipsoids with perpendicular axes. The ellipsoid with higher eccentricity is the best possible candidate site for whiskers. These whiskers are then traced in every single frame of the video sequence by using expectation maximization. The approach has some strong points. It requires no manual initiation, it is highly accurate and because of superb spatial resolution and pixel-level tracking, even micro-movements of whiskers can be tracked. But all the strengths come at a cost; the approach is computationally very expensive which means it cannot be deployed in real-time. There is another downside to pixel-level and frame-level processing, the temporal context is lost in the process.

Heidi et al. [109], proposed Automated Gait Analysis Through Hues and Areas (AGATHA). AGATHA first isolates the sagittal view of the animal by subtracting a background image where the animal is not present, transforming the frame into an HSV (Hues, Saturation, Value) image. The hue values are used to convert the HSV image into a binomial silhouette. Next, AGATHA locates the row of pixels representing the interface between the rat and the floor. AGATHA may not accurately locate the rat-floor interface if the animal moves with a gait pattern containing a completely aerial phase. Second, AGATHA excludes the majority of nose and tail contacts with the floor by comparing the contact point to the animal’s center of the area in the sagittal view. Foot contact with the ground is visualized over time by stacking the rat/floor interface across multiple frames. The paw contact stacked over multiple frames is then used for gait analysis. Multiple gait parameters such as limbs velocity, stride frequency can be calculated. When results from AGATHA were compared to manual annotation on a 1000 fps video, they deviated by a small amount. For example, limbs velocity calculated b AGATHA was 1.5% off from the velocity calculated manually. Similarly, AGATHA registered a difference of 0.2 cm in stride length from the manual annotation. This approach is simple and scalable and can be easily exported to track gait parameters from other angles of view. However, the gait parameters calculations rely on the subject contact with the floor, therefore, it might not be able to calculate the gait parameters in an aerial pose, therefore, this approach is limited in scope.

#### 7.2.2. Statistical/Learning-Based Approaches

Dankert et al. [110] proposed a machine vision-based automated behavioral classification approach for Drosophila. The approach does not cover locomotion in rodents, it covers micro-movements in flies. Videos of a pair of male and female flies are recorded for 30 min in a controlled environment. Wingbeat and legs motion data is manually annotated for lunging, chasing, courtship and aggression. The data analysis consists of four stages. In the first stage, the Foreground image $F_{I}$ is computed by dividing the original image I by ($\mu I+3\sigma_{I}$) ($F_{I}$ values in false-colors). In the second stage, The fly body is localized by fitting a Gaussian mixture model [111] (GMM) with three Gaussians; background, other parts, and body to the histogram of $F_{I}$ values (gray curve) using the Expectation-Maximization (EM) algorithm [111]. First (top) and final (bottom) iterations of the GMM-EM optimization. All pixels with brightness values greater than a threshold are assigned to the body and are fitted with an ellipse. In the third stage, the full fly is detected by segmenting the complete fly from the background, with body parts and wings [112]. In the fourth stage, head and abdomen are resolved by dividing the fly along the minor axis of the body ellipsoid and comparing the brightness-value distribution of both halves. In the fifth stage, 25 measurements are computed, characterizing body size, wing pose, and position and velocity of the fly pair. A k-nearest neighbor classifier is trained for action detection. The authors have reported a false positive rate for lunging at 0.01 when 20 min worth of data was used for training the classifier. Although this article does not directly deal with rodents, the detection and tracking algorithms used for legs and wings can be used for legs motion detection in rodents too. The approach is built upon proven statistical models. It can handle instrument noise. Since it has a learning element, the more data it sees, the better it gets. However, since the learning is not end to end, and the data processing pipeline is complex and need in depth understanding of statistical theory, therefore, it is hard to work with for a neuroscientist. Since it has a background modeling component, therefore, it also suffers from the inherent weaknesses of background subtraction models i.e., sensitivity to sudden changes in environment.

Kim et al. [113] proposed a method similar to the one proposed by Clack et al. [108] to track whisker movements in freely behaving mice. They use Otsu’s algorithm to separate foreground and background and then find the head of the mouse by locating a triangular-shaped object in the foreground. Once the head and snout are detected, the Hough transform is used to find line-like shapes (whiskers) on each side of the snout. Midpoints of the detected lines are used to form ellipsoidal regions which help track whiskers in every single frame. This pipeline was proposed to track whisking in mice after a surgical procedure. There is no ground truth available, so the approach cannot be evaluated for tracking quantitatively. The pipeline is simple and easy to follow. It can be used to track heads and whiskers in freely behaving mice. However, it is not feasible for real-time deployment due to high computational costs

Palmer et al. [114] proposed a paw-tracking algorithm for mice when they grab food and can be used for gesture tracking as well. They developed the algorithm by treating it as a pose estimation problem. They model each digit as a combination of three phalanges (bones). Each bone is modeled by an ellipsoid. For 4 digits, there is a total of 12 ellipsoids. The palm is modeled by an additional ellipse. The forearm is also modeled as an ellipsoid while the nose is modeled as an elliptic paraboloid. The paw is modeled using 16 parameters for the digits (four degrees of freedom per digit), four constant vectors representing the metacarpal bones and 6 parameters for position and rotation of the palm of the paw. Furthermore, the forearm is assumed to be fixated at the wrist and can rotate along all three axes in space. This amounts to a total of 22 parameters. In each frame, these ellipsoids are projected in such a way that they best represent the edges. The best projection of ellipsoids is found by optimization and is considered a paw. They haven’t reported any quantitative results. This approach is very useful if the gesture tracking problem is treated as pose estimation with a temporal context. Since the approach treats gesture tracking as a pose-estimation problem, it opens the possibility of using state-of-the-art pose-estimation methods in gesture tracking. However, the computational cost is high for real-time deployment without graphical accelerators.

In [115], Palmer et al. extended their work from [114]. The basic idea is the same. It models the paw made of different parts. Four digits (fingers), each digit having 3 phalanges (bones). Each phalange is modeled by an ellipsoid, so there is a total of 12 ellipsoids for the phalanges plus an additional one for the palm. In this paper, the movement of the 13 ellipsoids is modeled by vectors with 19 degrees of freedom, unlike 22 from [114]. The solution hypothesis is searched not simultaneously, but in stages to reduce the number of calculations. This is done by creating a different number of hypotheses for every joint of every digit and then finding the optimum hypotheses.

A Giovannucci et al. [116] proposed an optical flow and cascade learners-based approach for tracking of head and limb movements in head-fixed mice walking/running on a spherical/cylindrical treadmill. Unlike other approaches, only one camera installed from a lateral field of view was used for limb tracking and one camera installed in front of the mouse was used for whisker tracking. They calculated dense optical flow fields in a frame-to-frame method for whisker tracking. The estimated optical flow fields were used to train dictionary learning algorithms for motion detection in whiskers. They annotated 4217 frames for limb detection and 1053 frames for tails detection and then used them to train Haar-Cascades classifiers for both the cases. They have reported a high correlation of 0.78±0.15 for whiskers and 0.85±0.01 for hind limb. The proposed hardware solution in the paper is low cost and easy to implement. The tracking approach is also computationally not demanding and can be run in real-time. They, however, did not deal with the micro-patterns in motion dynamics which can be best captured with the inclusion of temporal context to the tracking approach. Moreover, accurate estimation of flow fields either takes too much time or requires graphical processing units.

Mathis et al. [117] introduced a user-defined body-parts tracking method based on deep learning called DeepLabCut. The body-part (which can be either limbs or tail or head) is built on top of the human pose estimation method based on deep learning called DeeperCut [118]. The DeepLabCut employs the feature detectors of DeeperCut to build user-defined body part detectors in laboratory animals. The training procedure is standard, a user manually annotates limbs/tail/body parts in some of the video frames which are used to fine-tune the DepperCut feature detectors. Then another prediction layer predicts the pose of the animal by labeling the body parts in question. The authors have reported accuracy of 4.17±0.32 pixels on test data. The reported architecture is remarkable since it is a general-purpose architecture and can be modified to track another body part relatively easily. Also, since the architecture is built upon existing state-of-the-art deep networks for pose estimation, it is easy to train and inherits all the strengths of parent deep networks. However, the reported pipeline can have a problem in case of occlusions.

In DeepBehavior, the authors have proposed an open-source behavioral analysis toolbox built on top of existing validated approaches [119]. The toolbox contains routines for gesture tracking, 3D kinematics analysis for humans and rodents and behavioral analysis for rodents. The toolbox is built on top of three existing and validated convolutional neural networks architecture named Tensorbox [120], YOLOv3 [121], and Openpose [122]. For 3D kinematics tracking, the toolbox needs a stereo system with a properly calibrated camera. They recommend to use Tensorbox if only one test subject needs to be tracked, YOLOv3 if multiple test subjects need to be tracked and Openpose if human subjects need to be tracked. They have initialized the networks by using models trained on ImageNet and fine tuned them with custom datasets. The authors have not provided paw tracking results for rodents. This toolbox is a good example of using gesture and pose tracking approaches developed and tested for humans to be used for rodents and small animals. Since the system is built upon existing state-of-the-art pose estimation architectures, it inherits their strengths and weaknesses. For instance, Openpose can have a hard time identifying a pose it has not seen. It also does not know how to tell two subjects apart, therefore, it can try to impose one pose upon two test animals in a situation in which one animal is partially occluded by the other. Also, it can face difficulties in estimating pose of animals at an angle.

## 8. Applications

Gesture tracking is finding applications not only in behavioral research but in other fields of research as well. For instance, researchers working in neurophysiology can benefit greatly from a dataset in which they can link brain patterns to a certain physical activity. They can see how the body responds to certain brain injuries or gene mutations. If reliable gesture tracking methods are available, the researchers can analyze the response of test animals to external stimuli, treatment regimes, and brain injuries. Moreover, researchers working on physiotherapies can benefit from gesture tracking. To sum it up, researchers working in one of the following fields may find some of the methods described in this survey useful for their purpose:

1. Research on behavioral phenotyping needs huge volumes of annotated data to understand and classify rodents’ and animals’ behaviors. By looking at the current state-of-the-art of gesture tracking/pose estimation methods, a researcher working on behavioral phenotyping can choose the gesture tracking/pose estimation method most suitable to their needs [6].
2. Research on depression analysis in normal and transgenic mice/animals can also benefit from this survey because for reliable quantification of depression, the researcher needs to understand the mice/animals behavior and once they have an appropriate quantification of behavior in terms of pose, locomotion, and gait patterns, they can understand how this behavior changes in response to genetic mutations. Scientists can now breed genetically-altered mice called "transgenic mice" that carry genes that are similar to those that cause human diseases. Likewise, select genes can be turned off or made inactive, creating "knockout mice," which can be used to evaluate the effects of cancer-causing chemicals (carcinogens) and assess drug safety, according to the FBR [123,124,125,126,127,128,129].
3. Researchers working on anxiety in rats/small animals can also benefit from the methods described this survey because with a suitable gesture tracking/pose estimation method, they can quantify rats/animals behavior efficiently and understand how it alters with anxiety [130,131,132,133].
4. Research on the effects of drugs and cancer on locomotion can also benefit from the methods described in this survey as gesture tracking/pose estimation can be used to understand the changes in locomotion patterns of rats/animals in response to tumors and drugs [134,135,136,137,138,139,140,141,142].
5. Another field which can benefit from automated gesture analysis is the research on understanding how neural activity controls physical activity or how the brain responds to external stimuli [143,144,145,146,147].
6. Research on neurophysiological and physical therapies can also benefit from the methods described in this survey. If a researcher can quantify the changes in gait patterns and pose over an extended time, he/she can track the success of therapy on recovery [148,149,150,151,152].
7. Researchers working on systems biology can also benefit from the methods described in this survey as with a proper gesture/gait analysis method. They can provide useful numerical evidence to understand the behavior of biological systems under different physical and pathological conditions [153].

## 9. Conclusions

In this paper, we provide a comprehensive survey of the main approaches for gesture tracking on small rodents, although we did not restrict the review to papers dealing with rodents but included related works that could be ported to the field. We included in Appendix A a complete summary of the approaches selected in this survey, with a special focus on the main characteristics of each paper (code availability, performance measures, setup, and invasiveness).

Gesture detection and tracking approaches are still in the developing phase. There is no single approach strong enough which can track micro-movements of limbs, whiskers or snouts of the rodents which are necessary for gesture identification and behavioral phenotyping. In general, those approaches which use specialized hardware are more successful than those approaches which solely depend on standard video cameras. For example, the use of X-Ray imaging to detect surgically implanted markers has been proven very successful in tracking limb and joint movements with high precision. Moreover, the use of specific markers attached to either limbs or whiskers of the rodents also increases the overall tracking accuracy of an approach. However, there is a downside to this approach; the rodents might not behave naturally. Therefore, more and more research is being conducted on scalable, portable and noninvasive tracking methods that only need standard video cameras.

### Future Research

Based on the literature survey we conducted, we have the following recommendations for future research:

1. Methods would benefit from the effective use of different camera configurations to get spatial data at high resolution in 3D space. Until now, only low-resolution time-of-flight sensors [93,97]. Acquiring spatial data from high-resolution cameras will help understand the gait of rodents in 3D.
2. One of the most relevant shortcomings of the field is the lack of public databases to validate new algorithms. Different approaches are tested on the (usually private) data from the lab developing the solution. Building a standardized gesture tracking dataset which can be used as a benchmark would similarly benefit the community as large object recognition databases (PASCAL, ImageNet or MS COCO) allowed significant progress in the Computer Vision literature.
3. Currently, large amounts of non-labeled data samples are in existence (thousands of video hours). The use of unsupervised learning algorithms that could benefit the parameter learning of supervised methods is one of the most challenging future research lines. Since unsupervised and weakly supervised gesture tracking/pose estimation is being researched for other species, extending it to rodents/small animals will make the large volumes of unlabeled data useful [154,155,156,157,158,159].
4. Data augmentation using synthetic samples. Now, methods based on GANs are obtaining extraordinary results in Computer Vision. Using GAN networks can help generate large amounts of annotated training data. The annotated data can then be used to validate gesture tracking/pose estimation techniques for rodents/small animals and those techniques can be further fine-tuned by a small set of human-annotated data.
5. Combine hardware-based methods with markers to create large scale databases for further automated learning just from the image. Up until, physical markers and specialized hardware have been used only in specific settings. They can be used to generate large volumes of annotated data by careful data acquisition as the markers can be reliably tracked by specialized hardware.
6. Besides, the use of semi-supervised and weakly-supervised learning algorithms could benefit the community. The challenge in this particular case is to minimize the user intervention (supervision) maximizing the improvements on the accuracy.
7. Very few of the surveyed approaches in the software-based method section consider temporal coherence while developing a solution for gesture tracking/pose estimation of rodents and small animals. Since locomotion is temporally coherent, machine learning methods such as Long Short Term Memory networks can be efficiently trained to track the rodents’ pose by evaluating the specific pose history.
8. Finally, deep learning methods have been shown to outperform many computer vision tasks. For instance, deep learning-based methods for gesture tracking/pose estimation in humans. Exploring these validated approaches can increase the reliability of gesture tracking/pose estimation in rodents/small animals [160,161].

## Appendix A. Summary of Selected Approaches

We have summarized some important aspects of selected approaches in Table A1. We use the following notation to properly interpret the table:

Code availability

It means whether the code is available or not. If it is available, is it free or paid.

Performance:

If the performance is given in terms of standard deviation, it signifies the consistency of proposed approach either against itself or an annotated dataset (which is pointed out). For example, if the table says that the proposed system can make a 90% accurate estimation of limbs velocity with an SD of 3%, it means that the system performance fluctuates somewhere between 87% to 93%. If absolute accuracy is given, it means each and every detected instant is compared to manually annotated samples. If only % SD is given or just SD is given, it means that the system can consistently reproduce the same result with specified amount of standard deviation, regardless of its performance against the ground truth.

Need specialized setup & Invasiveness

This indicates whether the method needs any specialized hardware other than the housing setup or video cameras. If the housing setup itself is arranged in a specific way but it does not contain any specialized materials, we say that the hardware setup required is not specialized. By invasiveness, we mean that a surgery has to be conducted to implant the markers. If no surgery is needed to implant markers, we call it semi-invasive. If no markers are needed, we call it non-invasive.

**Table A1:** Comparison of different approaches. Legend:: Invasive: Approaches which requires surgery to put markers for tracking, semi-invasive: Approaches which do not need surgery for marker insertion, non-invasive: no marker needed. Real time means that the system can process frames at the same rate they are being acquired. If it needs specialized equipment apart from standard video cameras and housing setup, it is pointed out in the last column.

	**Type**	**Code Availability**	**Performance**	**Real Time or Offline**	**Need Specialized Setup** & **Invasiveness**
[59]	Commercial	Paid	Comparison with ground truth not provided. One paper reports the reproducibility: 2.65% max SD	Yes	Yes
[71]	Commercial	Paid	Comparison with ground truth not provided. One paper reports the reproducibility: 1.57% max SD	Yes	Yes
[49]	Research	data and code for demo available at http://bit.do/eTTai	tracking performance not reported, behavioral classification of 12 traits reported to be max at 71%	Tracking real time, classification offline	yes
[51]	Research	not available	tracking: SD of only 0.034% when compared with ground truth, Max SD of 1.71 degrees in estimating joint angle	real time legs and joints tracking	yes, invasive
[52]	Research	not available	tracking performance not reported explicitly	real time whisker tracking	yes, semi-invasive
[53]	Research	available on request	whisker tracking performance not reported explicitly	real time single whisker tracking	yes, semi-invasive
[54]	Research	not available	head motion tracked correctly with a max false positive of 13%	real time head and snout tracking	yes, semi-invasive
[55]	Research	not available	head motion tracked continuously with a reported SD of only 0.5 mm	real time head and snout tracking	yes, semi-invasive
[57]	Research	not available	head motion tracked with an accuracy of 96.3% and the tracking can be reproduced over multiple studies with a correlation coefficient of 0.78	real time head tracking	yes, semi-invasive
[89]	Research	code and demo data available at https://goo.gl/vYaYPy	they reported a correlation between whisking amplitude and velocity as a measure of reliability, R = 0.89	Offline head and whisker tracking	no, invasive
[90]	Research	not available	Tracking and gait prediction with confidence of 95%, deviation between human annotator and computer at 8%	Offline	yes, semi-invasive
[93]	Research	not available	Paw tracked with an accuracy of 88.5 on transparent floor and 83.2% on opaque floor	Offline	yes, semi-invasive
[95]	Research	code available at https://goo.gl/58DQij	tail and paws tracked with an accuracy >90%	Real time	yes, semi-invasive
[97]	Research	not available	5 class behavioral classification problem, accuracy in bright condition is 95.34 and in dark conditions is 89.4%	offline	yes, non-invasive
[100]	Research	not available	6 behavioral class accuracy: 66.9%, 4 behavioral class accuracy: 76.3%	offline	yes, non-invasive
[98]	Research	code available at https://goo.gl/eY2Yza	whisker detection rate: 76.9%, peak spatial error in whisker detection: 10 pixels	offline	yes, non-invasive
[101]	Research	not available	Peak deviation between human annotator and automated annotation: 0.5 mm with a camera of 6 pixel/mm resolution	offline	yes, non-invasive
[92]	Research	not available	Tracking accuracy >90% after the algorithm was assisted by human users in 3–5% of the frames	offline	yes, semi-invasive
[105]	Research	code available at https://goo.gl/Gny89o	A max deviation of 17.7% between human and automated whisker annotation	offline	yes, non-invasive
[107]	Research	not available	Maximum paw detection error: 5.9%, minimum error : 0.4%	offline	no, non-invasive
[110]	Research	Source code at https://goo.gl/zesyez, demo data at https://goo.gl/dn2L3y	Behavioral classification: 1% false positive rate	offline	no, semi-invasive
[108]	Research	Source code available at https://goo.gl/JCv3AV	Whisker tracing accuracy: max error of 0.45 pixels	offline	no, non-invasive
[116]	Research	not available	Correlation with annotated data; for whiskers r = 0.78, for limbs r = 0.85	real time	no, non-invasive
[109]	Research	code available at https://goo.gl/V54mpL	Velocity calculated by AGATHA was off from manually calculated velocity by 1.5%	real time	no, non-invasive
[117]	Research	code available at http://bit.ly/2vgJUbr	Detected pose matched ground truth with an accuracy of 4.17±0.32 pixels	real time on GPUs	no, non-invasive
[119]	Research	code available at https://bit.ly/2XuJmPv	No performance metric reported	offline	no, non-invasive
